# Obstructive Ventilatory Defects in Pulmonary Arterial Hypertension and Chronic Thromboembolic Pulmonary Hypertension

**DOI:** 10.3390/jcm15218574

**Published:** 2026-11-03

**Authors:** Torben Sonneck, Michaela Barnikel, Paola Arnold, Gabriela Leuschner, Amanda Tufman, Jürgen Behr

**Affiliations:** 1 Department of Medicine V, LMU University Hospital, LMU Munich, Comprehensive Pneumology Center (CPC-M), Member of the German Center for Lung Research (DZL), 81377 Munich, Germany; 2 Division of Rheumatology and Clinical Immunology, Department of Medicine IV, LMU Medizin, LMU Munich, 80336 Munich, Germany

**Keywords:** pulmonary arterial hypertension, chronic thromboembolic pulmonary hypertension, obstructive ventilatory defects

## Abstract

**Background/Objectives:** Patients with pulmonary arterial hypertension (PAH) may display obstructive ventilatory defects outside of defined obstructive lung disease. However, its pathogenesis and prognostic relevance remain unknown. Similar data have emerged for patients with chronic thromboembolic pulmonary hypertension (CTEPH), but there is no description in a real-world European cohort. **Methods:** We performed a single-center, retrospective, cross-sectional study of patients with PAH (*n* = 46) and CTEPH (*n* = 45) at an academic PH center. Data were collected on pulmonary function tests, DLCO, right heart catheterization results, pre-existing diagnoses of asthma and chronic obstructive pulmonary disease (COPD), current medications, and adverse outcomes. **Results:** Patients with PAH and CTEPH showed a reduced FVC (81% for both groups), FEV_1_ (74 and 75%) and DLCO (54 and 53%). A previous diagnosis of COPD was documented in 11% of patients, and asthma in 11% of patients with PAH and 4.5% of patients with CTEPH respectively. Even after excluding patients with asthma or COPD, ventilatory obstruction was still present in at least 22% of patients and 16% were being treated with long-acting bronchodilators or inhaled corticosteroids. Of these patients, those who had adverse outcomes (active listing for lung transplantation, lung transplantation or death) had a significantly lower FVC (68 vs. 83%, *p* = 0.026), FEV_1_ (61 vs. 79%, *p* = 0.01) and MEF_50_ (38 vs. 59%, *p* = 0.021). All patients with adverse outcomes had a mean expiratory flow at 50% vital capacity (MEF_50_) < 60%, as opposed to 57% of those without an adverse event, however MEF_50_ did not predict adverse outcomes after adjusting for FVC. **Conclusions:** Ventilatory obstruction is common in patients with PAH and CTEPH even when excluding patients with asthma/COPD or a history of smoking. Symptomatic treatment with bronchodilators is common even without these comorbidities. Patients with adverse outcomes had lower FEV_1_, FVC and MEF_50_, but the functional relevance of the latter remains to be explored.

## 1. Introduction

Pulmonary hypertension (PH) is a heterogeneous and often fatal disease of the pulmonary vasculature that can present idiopathically, due to chronic thromboembolism, or in association with other systemic diseases. Typical signs and symptoms of PH like exertional dyspnoea, right heart failure with edema, hypoxemia and reduced diffusion capacity are mostly nonspecific, which can lead to considerable delay in diagnosis.

Pulmonary arterial hypertension (PAH, Group 1) is characterized by precapillary pulmonary hypertension on right heart catheterization in the absence of other causes of raised pulmonary-arterial pressure, such as chronic thromboembolic pulmonary hypertension (CTEPH, Class 4.1) or pulmonary hypertension due to left heart disease (Group 2) or lung disease (Group 3). CTEPH is defined as pulmonary hypertension in the context of past pulmonary embolism, with imaging demonstrating chronic pulmonary embolism and typical ventilation/perfusion mismatch despite adequate anticoagulation [1].

It has been described that some degree of ventilatory obstruction on lung function testing is common in patients with idiopathic or hereditary pulmonary arterial hypertension [2]. This was also found in patients with PH associated with congenital cardiac defects or connective tissue disease [3]. Studies on the frequency of ventilatory obstruction in chronic thromboembolic pulmonary hypertension have only appeared more recently. One study in a Russian cohort found that 23% of patients with CTEPH who were referred for pulmonary endarterectomy met the criteria for a diagnosis of COPD and two studies in Japanese cohorts found a prevalence of obstruction in CTEPH patients between 25 and 31% [4,5,6]. There have been associations between obstruction on spirometry and radiographic measures of vascular involvement in both PAH and CTEPH [6,7]; however it remains unclear which degree of ventilatory obstruction can be attributed to vascular involvement, or obstructive airway diseases like asthma or COPD.

In this retrospective, cross-sectional study we reviewed the clinical data of patients with PAH and CTEPH attending the pulmonary hypertension outpatient clinic at the LMU Munich University Hospital to assess the prevalence of ventilatory obstruction (defined as an FEV_1_/FVC below the lower limit of normal), elevated airway resistance or a reduced MEF_50_ as a marker of small airway disease, as well as documented comorbidities, markers of allergic and eosinophilic inflammation and outcomes like death or transplantation, to give a thorough description of ventilatory abnormalities and comorbidities in this population and generate hypotheses for potential biomarkers of adverse prognosis.

## 2. Materials and Methods

For the population, we retrospectively reviewed the clinical data of all patients who attended our PH center from February 2019 to March 2020. We included patients with pulmonary hypertension confirmed by right heart catheterization and a documented clinical diagnosis (according to the 2022 classification criteria) of either:

- Pulmonary arterial hypertension (“PAH”, Group 1) due to:
  ○ Idiopathic pulmonary hypertension (Group 1.1);
  ○ Hereditary pulmonary hypertension (Group 1.2);
  ○ Pulmonary venoocclusive disease (Group 1.5);
- Chronic thromboembolic pulmonary hypertension (CTEPH, Group 4.1).

Patients with other types of pulmonary hypertension especially those with PH due to comorbid heart or lung disease (Groups 2 and 3) as well as patients with confirmed lung or airway disease other than asthma or COPD were excluded.

### 2.1. Parameters

Data was recorded for (source indicated in brackets):

- Type of pulmonary hypertension and specific therapy (chart review);
- Documented diagnosis of asthma or COPD (chart review);
- Documented history of smoking (chart review);
- Treatments with inhaled long-acting muscarinic antagonists (LAMAs), long-acting beta agonists (LABAs) and/or inhaled corticosteroids (ICSs) (chart review);
- Blood eosinophil count (laboratory reports);
- Total IgE levels (laboratory reports);
- Lung function testing including DLCO measurements (lung function reports);
- Mean pulmonary-arterial pressure (mPAP) and cardiac index (CI) on right heart catheterization (RHC) at first diagnosis (RHC reports/chart review);
- Lung transplantation, active listing for lung transplantation (chart review);
- Death (chart review, personal communication).

We used the following definitions for ventilatory abnormalities:

- FEV_1_/FVC < lower limit of normal (LLN) (“obstruction”);
- sRAW > upper limit of normal (>0.96 kPa·s for women or >1.18 kPa·s for men) (“raised airway resistance”);
- MEF_50_ < 60% of its predicted value (as a marker for small airway disease).

Adverse outcome was defined as any of the following:

- Active listing for lung transplantation;
- Undergoing lung transplantation;
- Death.

For chart review, all examinations, including lung function and laboratory tests, were performed as part of routine clinical visits according to the LMU University Hospital’s internal standards and while patients were receiving treatment according to the standard of care. Visits were regularly scheduled every 3 months, so in cases where multiple measurements were performed during the observation period the first value was recorded, unless stated otherwise.

For pulmonary function tests, all recorded values are pre-bronchodilator measurements. However long-acting bronchodilator treatment was not stopped for patients receiving it as part of their regular medication. If there was a visit during the observation period, during which body plethysmography, blood gas analyses and DLCO measurements were performed during the same visit, these measurements were recorded. If these examinations were performed during separate visits, the first of such measurements during the inclusion period was recorded for each parameter.

For right heart catheterization and hemodynamics, we recorded hemodynamic data from right heart catheterization on initial diagnosis. The data were collected either from catheter reports, if the first diagnosis was at our center, or taken from progress notes for patients who had received their first diagnosis elsewhere.

For death and transplantation, events were recorded if they occurred within the observation period, which limited the maximum follow-up to one year. However, there was no standardized follow-up duration. Active listing for lung transplantation and lung transplantation at our center were documented in patients’ chart. If patients died at our hospital this was also recorded in the patients’ chart. If a patient died out-of-hospital this was also recorded if we were informed about this by their next-of-kin.

### 2.2. Statistical Analyses

We analyzed the data in RStudio (Version 2023.12.1+402) using R (Version 4.5.1) and the tidyverse-suite (Version 2.0.0). The graphs were generated with ggplot2 (Version 3.5.2) and the tables and summary statistics, including *p*-values, were generated using the gt (Version 1.0.0) and gtsummary packages (Version 2.2.0).

Values for upper and lower limits of normal for FEV_1_, FVC, FEV_1_/FVC and DLCO were calculated using the ERS/GLI calculator Version 2.0 (available at https://gli-calculator.ersnet.org/ accessed on 20 June 2025) and using the reference values of the ERS global lung function initiative [8,9].

Missing values are stated in the corresponding tables. No inference for missing values was used.

### 2.3. Ethics Counseling for Retrospective Data Analysis

Initial approval for the retrospective analysis of data up to July 2019 was granted on 10 December 2019. Additional approval for retrospective data analysis for the entire observation period was granted on 10 December 2024. Due to the retrospective study design using patient data from routine clinical care the requirement for informed consent was waived in accordance with German data protection laws and the bylaws of the LMU medical faculty.

## 3. Results

### Study Cohort

**Figure 1:**
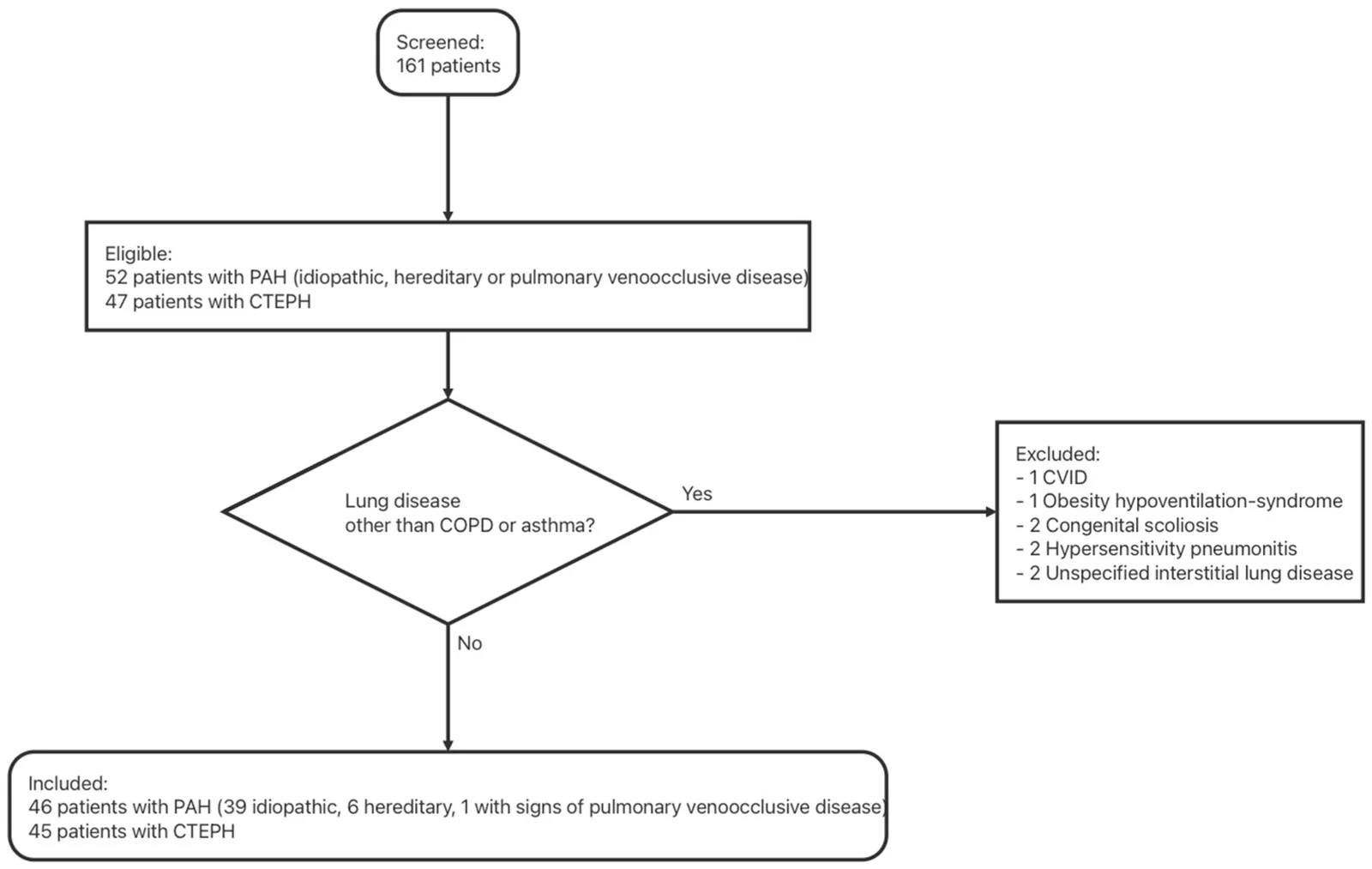
Screening and patient selection.

We screened 161 patients with PH and identified 52 patients with PAH (Group 1.1; 1.2 or 1.5) and 47 patients with CTEPH (Group 4.1). We excluded six and two patients respectively for lung diseases other than asthma or COPD (one CVID with bronchiectasis, one with obesity hypoventilation syndrome, two with congenital scoliosis, two with hypersensitivity pneumonitis, and two with unspecified ILD). The remaining 46 PAH and 45 CTEPH patients were included in the analysis (Figure 1).

Table 1 shows the baseline data for both diagnoses. Most patients were female (64% and 70% respectively). Patients with CTEPH were older, with a mean age of 71 years versus 53 years in those with PAH. Parameters for lung function and diffusion capacity for carbon monoxide (DLCO) were reduced to a comparable degree in both groups. A diagnosis of COPD was documented in 11% of patients in both groups, while a diagnosis of asthma was more common in PAH patients (11%) than in CTEPH patients (4.4%). A history of smoking was documented in 22% of patients with CTEPH and 11% of patients with PAH, although none of these differences were statistically significant. Until the end of the observation period six patients in the PAH group had died, and three had undergone lung transplantation, whereas in the CTEPH group only two patients had died, and none had undergone lung transplantation.

**Figure 2:**
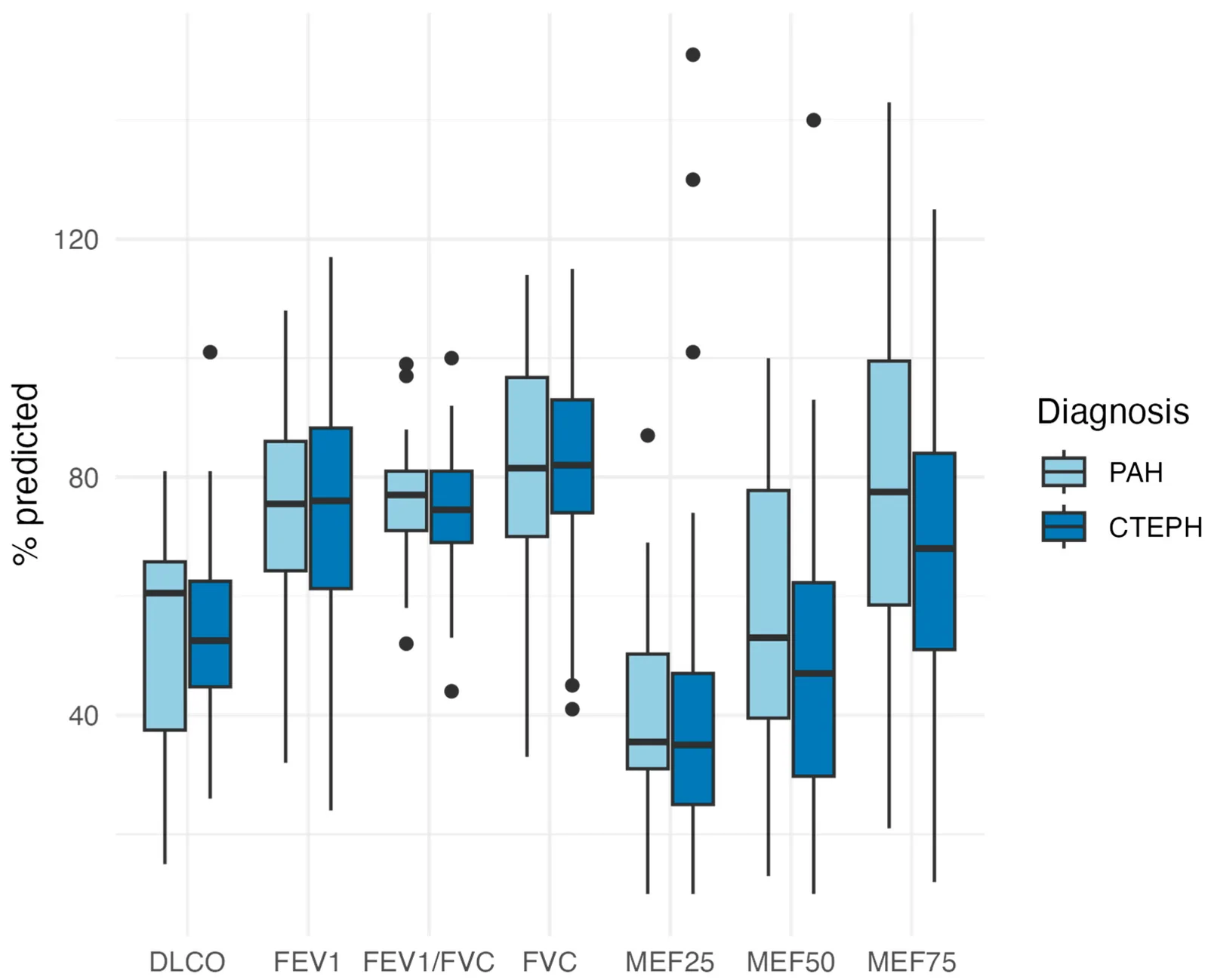
Lung function parameters in patients with PAH and CTEPH.

Figure 2 shows a comparison of the most important lung function parameters between patients with PAH and CTEPH. Most values were similar between the two groups, while DLCO and mean expiratory flow at 75% and 50% of FVC which were lower in patients with CTEPH.

Ventilatory abnormalities as defined above were common in both PAH and CTEPH patients. The prevalence tended to be higher in patients with CTEPH, although not significantly so (Table 2). FEV_1_/FVC below the lower limit of normal indicated obstruction in 22% and 25% of patients respectively, whereas both a specific airway resistance above the upper limit of normal and an MEF_50_ < 60% of its predicted value were present in more than half and up to 73% of the patients, which makes these markers less specific, but could represent a preferential affection of the small airways in this population [2].

We grouped and compared the patients based on a previously documented diagnosis of asthma or COPD and compared the groups regarding lung function, frequency of obstruction, RHC parameters and markers of eosinophilic inflammation as shown in Table 3.

As expected, ventilatory obstruction, raised airway resistance and a low MEF_50_ were significantly more common in patients with a diagnosis of asthma or COPD. But even patients with neither of these diagnoses showed obstruction in 16% of cases, and a raised airway resistance and low MEF_50_ in 64% and 60% of cases respectively. There were no significant differences in mPAP or DLCO between groups. A documented history of allergy was significantly more common in patients with asthma, and IgE levels tended to be higher in patients with a diagnosis of asthma or COPD, although this difference was not statistically significant. There was no relevant difference in the median blood eosinophil count.

Unsurprisingly, most patients with a diagnosis of asthma or COPD were being treated with a long-acting bronchodilator (LAMA or LABA) or an inhaled corticosteroid. Yet up to 16% of patients with neither of these diagnoses were receiving a long-acting bronchodilator or ICS. These patients had significantly lower FEV_1_ (79% vs. 62%, *p* = 0.02) and lower MEF_50_ (59% vs. 35%, *p* = 0.008) than those without inhaler therapy and were more likely to have an FEV_1_/FVC < LLN (43% vs. 11%, *p* = 0.06) (see Appendix A Table A1).

As an exploratory analysis we compared patients who had had an adverse outcome (defined as any of the following: death, active listing for lung transplantation or actual lung transplantation) and those without. To reduce confounding due to obstructive lung disease or smoking, we excluded all patients with a previous diagnosis of asthma and COPD or any documented history of smoking. The final number of patients for analysis was 62. Results are shown in Table 4.

Patients with an adverse outcome had significantly lower FVC, FEV_1_ and MEF_50_. All patients with adverse outcomes had an MEF_50_ < 60%, as compared with 57% of those without an adverse outcome. However, after adjusting MEF_50_ for FVC (by dividing MEF_50_ by FVC as % predicted), the difference was no longer statistically significant. There were no significant differences in other markers of obstruction.

## 4. Discussion

The mechanism by which pulmonary arterial hypertension might be linked to ventilatory obstruction is not clear, although there have been various hypotheses in the past such as mechanical compression by thickened and dilated vessels, raised intrathoracic blood volume and altered levels of inflammatory or vasoactive mediators [7,10,11,12,13,14,15,16]. It is also not clear whether impaired lung function and obstruction are merely epiphenomena of vascular changes or might contribute to morbidity and mortality themselves.

The overall prevalence of obstruction of 22–25% in our patient sample (as defined by an FEV_1_/FVC < LLN) is consistent with previous studies [2,4,5,6]. The prevalence of 10% for a diagnosis of asthma and COPD respectively is consistent with the prevalence in the general population in Germany, with 8% for asthma and 8.5% for COPD in people aged 40 and older [17]. This indicates that there is no over- or underrepresentation of these diagnoses in patients with CTEPH or PAH.

Yet, even after excluding patients with previous diagnoses of asthma and COPD or any recorded history of smoking there remains a sizeable subset of patients with obstruction, which cannot be readily explained by other means. Although conclusive testing for bronchial hyperreactivity or bronchodilator responsiveness was lacking, other studies have found similar rates of bronchial obstruction when excluding all patients with a smoking history or any bronchodilator treatment [2,3,4,5]. Also, our data showed no elevations in markers of eosinophilic inflammation or allergy, which might imply undiagnosed asthma.

Unraveling the cause of obstruction is further complicated by the fact that airway obstruction and dyspnoea in PAH partially respond to short-acting bronchodilator treatment, which could lead to overdiagnosis of asthma or COPD in these patients [18,19]. As our data show, many patients receive symptomatic treatment with long-acting bronchodilators without a formal diagnosis of asthma or COPD. Treatment with short-acting bronchodilators was not recorded, and is presumably even more common. Some studies have shown the presence of dynamic hyperinflation in patients with PAH despite normal lung function at rest [20,21] and studies in patients with COPD have shown that reducing hyperinflation with long-acting bronchodilators can lead to improved cardiac function due to improved diastolic filling [22]. Given that treatment with short-acting bronchodilators in PAH patients improved hemodynamic variables in the short term [18], it seems possible that long-acting bronchodilators could also positively affect these variables, although there are currently no studies to support their use in these patients, and further clinical studies are needed.

Inhaled treatments for PH have shown mixed results and most studies either did not assess spirometry at baseline or did not stratify patients based on obstruction on spirometry [23,24,25,26,27,28]. This might provide further clues about the interaction of PH and other lung diseases, especially obstructive pulmonary disease.

While oral imatinib, a tyrosine-kinase inhibitor, lowered pulmonary vascular resistance, a phase 2b trial of inhaled imatinib showed no effect on pulmonary vascular resistance [23,24]. Inhaled Iloprost, a prostaglandin analog, improved the 6 min walking distance (6-MWD) in patients with severe pulmonary hypertension and the INCREASE trial showed an increase in 6-MWD with inhaled Treprostinil in patients with pulmonary hypertension associated with interstitial lung disease (PH-ILD), leading to FDA approval for this indication [25,26]. The proposed mechanism of action is that the inhaled medication is preferentially delivered to well-ventilated areas of the lung, where the vasodilatory effect reduces ventilation/perfusion mismatch and thus increases gas exchange. Notably, a trial of inhaled Iloprost in pulmonary hypertension associated with COPD (COPD-PH) showed no benefit, and the PERFECT trial of inhaled Treprostinil in patients with COPD-PH was terminated early due to safety concerns in the treatment group [27,28]. In a post-hoc analysis of the PERFECT trial an FEV_1_ of >40% in patients with an mPAP of >40 mmHg emerged as a predictor of treatment response, which highlights the potential of severe obstruction to interfere with the efficacy of inhaled treatments in pulmonary hypertension and the need for future studies to assess the degree of obstruction at baseline. It is also not known if concomitant or sequential treatment with a bronchodilator affects the delivery or efficacy of other inhaled medications for pulmonary hypertension.

A 2002 study showed significant differences in lung function tests when comparing PAH patients with an mPAP of >56 mmHg and those with lower pressures on right heart catheterization [2]. Patients with a very high mPAP had lower FVC, FEV_1_ and peak expiratory flow and a higher residual volume. MEF_50_ was decreased, but similar in both groups. However, this study did not include data on outcomes. Another study by Rahaghi et al. [7], showed that various parameters of lung function, especially obstruction, worsened over the years between initial diagnosis and eventual transplantation in patients with advanced pulmonary hypertension. Losses in lung function correlated most closely with intrathoracic blood volume and enlargement of the pulmonary arteries without other explanations for the obstruction on pathologic examination of the explanted lungs. Notably there were no significant differences in hemodynamic variables between the first diagnosis and eventual transplantation. One study in CTEPH showed that increased vascular obstruction as measured by CT angiography correlated with lower FVC, FEV_1_, maximum mid-expiratory flow and MEF_50_, and improvements in vascular obstruction after pulmonary endarterectomy were correlated to improvements in FEV1 [6].

Our data showed significant differences in FVC, FEV_1_ and MEF_50_, when comparing patients with and without adverse outcomes, but not in DLCO, which is usually established as one of the most important prognostic parameters in pulmonary hypertension [29,30,31]. This could either be due to the small sample size, or due to selection bias in our cohort, as almost half of the adverse outcomes were related to lung transplantation and this probably introduces a bias towards younger patients with more advanced PH and fewer comorbidities, which might not be representative of other cohorts. Although MEF_50_ was significantly lower in patients with adverse outcomes and all patients with adverse outcomes had an MEF_50_ < 60%, this finding is not specific, as a reduced MEF_50_ < 60% was also present in 57% of patients without a negative outcome, and MEF_50_ did not differ significantly between these patients after adjusting for FVC. Whether markers of small airway obstruction like MEF_50_ or MMEF yield any additional prognostic information to more established parameters like FEV_1_, DLCO, or mPAP remains to be elucidated.

Overall, there is accumulating evidence for a stable association between vascular and hemodynamic changes and changes in lung function—most prominently obstruction—both as predictors of adverse outcomes, and as markers of improvement due to therapeutic interventions [2,6,7].

As for limitations, this study is limited by its retrospective and cross-sectional design and its small sample size for subgroups especially regarding adverse outcomes. Lung function tests and measurements of blood eosinophils and IgE were performed while some patients were receiving treatment with long-acting bronchodilators or inhaled corticosteroids, which may lead to an underestimation of obstruction and markers of eosinophilic inflammation in this subgroup. Also, data for bronchodilator responsiveness was not available, which leaves a possibility of confounding due to undiagnosed asthma or COPD. Follow-up duration and assessment for adverse events were not standardized, and follow-up was less than 1 year, which limits its value for prognostic analyses.

## 5. Conclusions

Our study adds a thorough description of lung function in a cross-section of patients with CTEPH and PAH. It provides further evidence that abnormalities of lung function, especially obstruction, are common in these patients, even when excluding those with asthma or COPD or a history of smoking. Symptomatic treatment with long-acting bronchodilators is common even in patients without a diagnosis of asthma or COPD, despite a lack of evidence to support these treatments, and further studies on their effect in PH might be warranted. Patients with adverse outcomes, such as lung transplantation or death, had significantly lower FVC, FEV_1_, and MEF_50_. Differences in other markers of obstruction or hemodynamic parameters did not reach statistical significance in our sample.

## Figures and Tables

**Table 1:** Baseline demographics and selected parameters.

Characteristic	PAH, *N* = 46	CTEPH, *N* = 45	*p*-Value ^1^
Age (years)	53 (18)	71 (14)	<0.001
Sex			0.6
Male	14 (30%)	16 (36%)	
Female	32 (70%)	29 (64%)	
BMI (kg/m^2^)	26.9 (5.5)	26.1 (6.2)	0.4
Time since diagnosis (years)	8.8 (6.2)	5.5 (3.8)	0.008
Asthma/COPD			0.6
Neither	36 (78%)	38 (84%)	
Asthma	5 (11%)	2 (4.4%)	
COPD	5 (11%)	5 (11%)	
History of smoking	5 (11%)	10 (22%)	0.14
FVC (% pred.)	81 (20)	81 (18)	>0.9
(Missing)		1	
FEV_1_ (% pred.)	74 (18)	75 (19)	0.8
(Missing)		1	
DLCO (% pred.)	54 (18)	53 (16)	0.5
(Missing)	4	1	
mPAP (mmHg)	42 (14)	39 (12)	0.3
Cardiac index (L/m^2^)	3.03 (0.89)	3.10 (0.76)	0.4
Treatment			
PDE5 inhibitors			<0.001
Sildenafil	23 (50%)	5 (11%)	
Tadalafil	12 (26%)	2 (4.4%)	
No PDE5i	11 (24%)	38 (84%)	
Endothelin receptor antagonists			0.012
Ambrisentan	9 (20%)	4 (8.9%)	
Bosentan	3 (6.5%)	1 (2.2%)	
Macitentan	24 (52%)	16 (36%)	
No ERA	10 (22%)	24 (53%)	
Riociguat	4 (8.7%)	33 (73%)	<0.001
Selexipag	10 (22%)	3 (6.7%)	0.040
Calcium channel blockers	6 (13%)	1 (2.2%)	0.11
Prostanoids			0.050
Intravenous Treprostinil	7 (15%)	2 (4.4%)	
Subcutaneous Treprostinil	1 (2.2%)	0 (0%)	
Inhaled Iloprost	2 (4.3%)	0 (0%)	
No Prostanoid	36 (78%)	43 (96%)	
Mortality during observation time			0.3
Alive	40 (87%)	43 (96%)	
Dead	6 (13%)	2 (4.4%)	
Transplantation			0.056
No transplantation	41 (89%)	45 (100%)	
Listed for transplantation	2 (4.3%)	0 (0%)	
Transplanted	3 (6.5%)	0 (0%)	

Mean (SD); *n* (%). ^1^ Wilcoxon rank sum test; Pearson’s Chi-squared test; Fisher’s exact test. BMI, Body mass index; FVC, forced vital capacity; FEV_1_, forced expiratory volume in 1 s; DLCO, diffusion capacity for carbon monoxide; mPAP, mean pulmonary-arterial pressure; ERA, endothelin receptor antagonist.

**Table 2:** Frequency of different respiratory abnormalities in patients with PAH or CTEPH.

Characteristic	PAH, *N* = 46	CTEPH, *N* = 45	*p*-Value ^1^
FEV_1_/FVC < LLN (Missing)	10 (22%) 0	11 (25%) 1	0.7
MEF_50_ < 60%	27 (59%)	32 (73%)	0.2
(Missing)	0	1	
sRAW > ULN	29 (63%)	32 (71%)	0.4

*n* (%). ^1^ Pearson’s Chi-squared test. FEV_1_, forced expiratory volume in 1 s; FVC, forced vital capacity; MEF_50_, mean expiratory flow at 50% FVC; sRAW, specific airway resistance; LLN, lower limit of normal; ULN, upper limit of normal.

**Table 3:** Lung function, RHC parameters and markers of allergy and eosinophilic inflammation, as well as LAMA/LABA or ICS use by previous diagnosis of asthma or COPD.

Characteristic	Overall, *N* = 91	Neither, *N* = 74	Asthma, *N* = 7	COPD, *N* = 10	*p*-Value ^1^
FVC (% pred.)	81 (19)	81 (19)	90 (11)	74 (22)	0.3
(Missing)	1	1	0	0	
FEV_1_ (% pred.)	75 (18)	77 (18)	74 (13)	60 (21)	0.080
(Missing)	1	1	0	0	
FEV1/FVC (% pred.)	75 (10)	78 (8)	70 (10)	62 (10)	<0.001
(Missing)	2	2	0	0	
FEV_1_/FVC < LLN	21 (23%)	12 (16%)	4 (57%)	5 (50%)	0.005
(Missing)	1	1	0	0	
MEF_50_ (% pred.)	53 (26)	57 (24)	44 (31)	27 (14)	<0.001
(Missing)	1	1	0	0	
MEF_50_ < 60%	59 (66%)	44 (60%)	5 (71%)	10 (100%)	0.023
(Missing)	1	1	0	0	
sRAW (kPa/s)	1.35 (0.60)	1.27 (0.49)	1.32 (0.55)	1.95 (1.02)	0.038
sRAW > ULN	61 (67%)	47 (64%)	4 (57%)	10 (100%)	0.042
DLCO (% pred.)	54 (17)	54 (17)	56 (14)	48 (16)	0.4
(Missing)	5	5	0	0	
mPAP (mmHg)	41 (13)	41 (14)	38 (10)	38 (14)	>0.9
IgE (U/mL)	58 (15, 287)	33 (10, 193)	96 (27, 248)	203 (79, 620)	0.14
(Missing)	46	42	0	4	
Blood eosinophil count (G/L)	0.13 (0.06, 0.18)	0.11 (0.06, 0.18)	0.12 (0.06, 0.24)	0.16 (0.13, 0.25)	0.3
(Missing)	14	13	0	1	
History of allergy	7 (7.7%)	3 (4.1%)	4 (57%)	0 (0%)	<0.001
LAMA or LABA	23 (25%)	12 (16%)	3 (43%)	8 (80%)	<0.001
ICS	13 (14%)	4 (5.4%)	6 (86%)	3 (30%)	<0.001

Mean (SD); median (Q1, Q3); *n* (%). ^1^ Kruskal–Wallis rank sum test; Fisher’s exact test. FVC, forced vital capacity; FEV_1_, forced expiratory volume in 1 s; MEF_50_, mean expiratory flow at 50% FVC; sRAW, specific airway resistance; ULN, upper limit of normal; DLCO, diffusion capacity for carbon monoxide; mPAP, mean pulmonary-arterial pressure; IgE, Immunoglobulin-E, LAMA, long-acting muscarinic agonist; LABA, long-acting beta agonist; ICS, inhaled corticosteroid.

**Table 4:** Comparison of patients with and without adverse outcomes (after excluding patients with COPD, asthma or a history of smoking).

Characteristic	No Adverse Outcome *N* = 55	Adverse Outcome *N* = 7	*p*-Value ^1^
Sex			>0.9
Male	16 (29%)	2 (29%)	
Female	39 (71%)	5 (71%)	
Age (years)	63 (20)	54 (12)	0.13
FVC (% pred.)	83 (20)	68 (11)	0.026
(Missing)	1	0	
FEV_1_ (% pred.)	79 (18)	61 (13)	0.010
(Missing)	1	0	
DLCO (% pred.)	55 (17)	51 (21)	0.7
(Missing)	3	2	
mPAP (mmHg)	41 (15)	47 (7)	0.10
Cardiac index (L/m^2^)	3.06 (0.78)	2.74 (0.55)	0.4
FEV_1_/FVC (%)	78 (8)	75 (7)	0.3
(Missing)	2	0	
FEV_1_/FVC < LLN	8 (15%)	1 (14%)	>0.9
(Missing)	1	0	
MEF_50_ (% pred.)	59 (25)	38 (12)	0.021
(Missing)	1	0	
MEF_50_ (FVC-adjusted)	72 (27)	57 (20)	0.3
(Missing)	1	0	
MEF_50_ < 60%	31 (57%)	7 (100%)	0.038
(Missing)	1	0	
sRAW (kPa/s)	1.24 (0.44)	1.47 (0.59)	0.4
sRAW > ULN	35 (64%)	4 (57%)	>0.9

*n* (%); mean (SD). ^1^ Fisher’s exact test; Wilcoxon rank sum test. FVC, forced vital capacity; FEV_1_, forced expiratory volume in 1 s; DLCO, diffusion capacity for carbon monoxide; mPAP, mean pulmonary-arterial pressure; FEV_1_, forced expiratory volume in 1 s; FVC, forced vital capacity; LLN, lower limit of normal, MEF_50_, mean expiratory flow at 50% FVC; sRAW, specific airway resistance; ULN, upper limit of normal.

## Data Availability

The data presented in this study are available on request from the corresponding authors. The data are not publicly available due to restrictions concerning privacy.

## Appendix A

**Table A1:** Ventilatory parameters and frequency of obstruction by treatment with a LAMA or LABA (after excluding patients with COPD, asthma or a history of smoking).

Characteristic	No LAMA or LABA, *N* = 54	LAMA or LABA, *N* = 8	*p*-Value ^1^
FEV_1_ (% pred.)	79 (18)	62 (15)	0.020
(Missing)	0	1	
FVC (% pred.)	82 (20)	71 (16)	0.12
(Missing)	0	1	
FEV_1_/FVC < LLN	6 (11%)	3 (43%)	0.059
(Missing)	0	1	
MEF_50_ (% pred.)	59 (24)	35 (18)	0.008
(Missing)	0	1	
MEF_50_ < 60%	32 (59%)	6 (86%)	0.2
(Missing)	0	1	

Mean (SD); *n* (%). ^1^ Wilcoxon rank sum test; Fisher’s exact test. FVC, forced vital capacity; FEV_1_, forced expiratory volume in 1 s; LLN, lower limit of normal, DLCO, diffusion capacity for carbon monoxide; mPAP, mean pulmonary-arterial pressure; FEV_1_, forced expiratory volume in 1 s; FVC, forced vital capacity; MEF_50_, mean expiratory flow at 50% FVC.
